# Economic and organizational impact of a clinical decision support system on laboratory test ordering

**DOI:** 10.1186/s12911-017-0574-6

**Published:** 2017-12-22

**Authors:** Elena Bellodi, Emidia Vagnoni, Barbara Bonvento, Evelina Lamma

**Affiliations:** 1 Department of Engineering, University of Ferrara, Via Saragat 1, Ferrara, Italy; 2 Department of Economics and Management and CRISAL, University of Ferrara, Via Voltapaletto 11, Ferrara, Italy; 3 Research Centre for the Health Care Economics and Management (CRISAL), University of Ferrara, Via Voltapaletto 11, Ferrara, Italy

**Keywords:** Clinical decision support system, Laboratory tests, Appropriateness, Repeat testing, Healthcare costs, End-user satisfaction, POESUS

## Abstract

**Background:**

We studied the impact of a clinical decision support system (CDSS) implemented in a few wards of two Italian health care organizations on the ordering of redundant laboratory tests under different perspectives: (1) analysis of the volume of tests, (2) cost analysis, (3) end-user satisfaction before and after the installation of the CDSS.

**Methods:**

(1) and (2) were performed by comparing the ordering of laboratory tests between an intervention group of wards where a CDSS was in use and a second (control) group where a CDSS was not in use; data were compared during a 3-month period before (2014) and a 3-month period after (2015) CDSS installation. To measure end-user satisfaction (3), a questionnaire based on POESUS was administered to the medical staff.

**Results:**

After the introduction of the CDSS, the number of laboratory tests requested decreased by 16.44% and costs decreased by 16.53% in the intervention group, versus an increase in the number of tests (+3.75%) and of costs (+1.78%) in the control group. Feedback from practice showed that the medical staff was generally satisfied with the CDSS and perceived its benefits, but they were less satisfied with its technical performance in terms of slow response time.

**Conclusions:**

The implementation of CDSSs can have a positive impact on both the efficiency of care provision and health care costs. The experience of using a CDSS can also result in good practice to be implemented by other health care organizations, considering the positive result from the first attempt to gather the point of view of end-users in Italy.

## Background

A clinical decision support system (CDSS) is a system designed to support clinical decisions during a diagnostic or therapeutic care process. When integrated with a computerized physician order entry (CPOE), CDSSs can guide a physician’s decisions during the process of entering medication orders or other physicians’ instructions. In particular, a CDSS can limit the repetition of redundant tests during the prescription of laboratory examinations (LEs). For example, the repetition of tests that have already been conducted can be avoided by suggesting an alternative plan of action. In a CDSS, a comment is required for a test (for example “Prole 20 is not needed more than once a day”) which other physicians can read before they override it and confirm their request (for instance, “clinical condition has changed” or “the last result requires confirmation”) [1].

Evidence shows that one of the causes of inappropriate ordering of laboratory tests is the prescription of tests earlier than necessary [2]. Recently, it has been estimated that between 6 and 20% of tests ordered, depending on the analyte, were inappropriate based on repeat criteria [3]. Zhi et al. conducted a systematic review and meta-analysis of laboratory testing in 2013, which covered a period of 15 years; they found that overutilization of repeat testing was 7.8% (95% CI 2.5-12.5%) for all analytes [4]. They demonstrated that repeat testing constitutes only a small portion of the overutilization of laboratory tests. They found that the overutilization rate of initial testing was much higher, at 44%, than that for repeat testing [4].

Over-ordering may be the result of inexperience, unnecessary diagnostic tests conducted in defensive medicine, inability to develop effective diagnostic planning because of inaccurate knowledge of a test’s properties, or the domino effect in which testing generates additional testing. Inappropriate LE repetition has several negative consequences: increase in costs, reduction of quality standards, and decrease in the definition of efficient therapies because of possible generation of false positives and psychological effects on patients. Different methods and forms of support are required to facilitate appropriate ordering, for instance medical involvement, incentives or economic penalties, administrative interventions, and educational measures [5–7] have been applied by professionals and scientific associations in an attempt to control the number of prescriptions of LEs. They have often been transient, or applicable only to a limited number of tests, or showed mixed results [8, 9].

What was shown to be effective in decreasing the number of redundant LEs was the use of computerized reminders. They can display to the physicians the date and result of the latest test and the likelihood of a positive result being a true positive. For many commonly ordered tests, intervals at which repeat testing is appropriate have been defined and accepted in the medical literature. These intervals can be used to show a warning at the time of ordering for inappropriate requests; physicians can override the reminder when entering a coded reason. The effects of CDSS implementation on the prescription of LEs were monitored and the results showed that tests were cancelled 69% of the time [10] with no significant mortality rate change.

The objective of our study was to evaluate the preliminary results from the implementation of a CDSS called PROMETEO in two Italian health care organizations located in the north-east of the Emilia Romagna region, and to study its impact on the prescription workflow, costs, tests volume, and job organization. Early results on the impact of the project on the amount of test ordering were published [11, 12].

## Methods

### Setting

The study was conducted in two public health care organizations: the Independent Hospital of Ferrara (IH) and two provincial hospitals (named Cento Hospital and Delta Hospital) belonging to the same local health authority (LHA) and located in the province of Ferrara. The IH is a teaching hospital with 637 ordinary hospital beds and 84 day hospital beds, serving a population of approximately 360,000 inhabitants. In 2015, there were 24,023 hospitalizations in ordinary beds and 8022 in day hospital beds. Cento and Delta Hospitals are two acute care local hospitals with 380 beds in total. Both the IH and the LHA hospitals have an integrated information system, accessed via networked desktop personal computers, which provides clinical and administrative functions. Laboratory tests are conventionally requested by the physician in charge of the patients using an order entry system (SAP at the IH and GALILEO at Cento and Delta Hospitals). In 2010, the IH requested approximatively 1,900,000 LEs for a total cost of 6 million euros, while the two LHA hospitals performed approximately 780,000 LEs for a total cost of 2.4 million euros. An initial analysis of the clinical database of the IH investigated three common examinations: thyroid-stimulating hormone (TSH), glycated hemoglobin (HbA1c), and antinuclear antibodies (ANA). The analysis showed a percentage of redundancy of 5% for TSH, 15% for HbA1c, and 16% for ANA, and that 60% of TSH, 71% of HbA1c, and 69% of ANA had been repeated prior to the optimal timing suggested by guidelines. The above-mentioned data motivated a debate about the need to change practice. Thus, since the beginning of 2014, a CDSS intervention was implemented full time in some wards, on a voluntary basis, of both the IH and the LHA hospitals. Considering the technological implications of the project, the inter-organizational departments of Information and Communication Technology (ICT) and the Provincial Laboratory of the health care organizations, the computer science company NoemaLife, and the Department of Engineering of the University of Ferrara were involved. The involvement of the University of Ferrara Research Center for Health Economics and Management (CRISAL) enabled assessment of the economic advantage resulting from the introduction of the CDSS, and the monitoring of inappropriate costs related to redundant laboratory requests.

### CDSS implementation and appropriateness rules

PROMETEO was implemented on top of an existing CPOE that handled requests for laboratory tests. It is an expert system able to manage LEs with high repetition probability (e.g., clinical chemistry), great complexity (e.g., autoimmunity and endocrinology), and can check for the presence of test requests that are mutually incompatible within the same order. Before the CDSS implementation, the existing CPOE allowed users to access the results of a patient’s previous tests only if explicitly requested; it did not automatically show the results of previous tests to the physicians at the time of ordering new tests. PROMETEO is able to verify the accuracy of orders and it is accessible from any requesting system outside of the Clinical Laboratory, showing the results automatically.

Its main technological characteristics are:

- Inferential engine based on the International Standard JSR-94. This allows immediate evaluation of rules and the order of the evaluation of the rules does not need to be defined a priori; rules can be configured to unambiguously describe experts’ knowledge.
- High degree of configurability. Graphical user interfaces (GUIs) can facilitate the setting of rules and the response messages that should be sent to the requesting systems connected to PROMETEO.
- Open source development environment.
- Fully scalable architecture. High performance to support high productivity environments.

Other innovative functionalities are:

- Independence from the order entry. PROMETEO can be activated from any internal or external requesting system.
- Rule monitoring. PROMETEO allows monitoring accesses from the requesting systems and the appropriateness verifications performed.
- Detailed summaries of the generated warnings can be requested.

PROMETEO is based on a three-layer architecture: UI Client Configuration, Application Server, Database Archive. The UI Client Configuration is used by the administrator to define appropriateness rules and to manage the definition and transcoding of examination. Thanks to a mapping mechanism, it is possible to define different rules for different types of requesting systems and to activate a similar rule on one or more connected systems.

The criteria defined to discover potentially redundant tests are based on both time and a quantitative evaluation of previous results. Table 1 shows an example of warnings of inappropriate tests based on time criteria.

**Table 1:** Warnings of inappropriate testing of analytes based on time criteria (aPTT: activated partial thromboplastin time, VANCO: vancomycin, CRP: C-reactive protein, PT: prothrombin time, FERRI: ferritin)

Analytes	Warning
aPTT	Test executed in the last 24h
VANCO	Test executed in the last 72h
Blood count	Test executed in the last 24h
CRP	Test executed in the last 72h
PT	Test executed in the last 24h
FERRI	This test should be repeated not before 3 months or as suggested by a specialist
TSH	This test should be repeated not before 6 months or as suggested by a specialist

Time criteria are defined based on the assumption that tests provide a different response after a given time interval (hours, days, months, years), whereas if done before the given time interval, the value of the result would not change. Therefore, the expert system is interested in computing the time elapsed between two sequential results for the same test: this difference is checked against a predetermined intersample time interval appropriate for that test according to the medical literature.

The quantitative evaluation of previous testing enables access to a past result’s value (for the class of examinations whose result is expressed by a numerical value). The definition of the normality intervals takes into account that multiple normality ranges can exist for a subset of the LEs, according to a patient’s sex and age. Each rule is described by testing-name, appropriate time interval between repetitions, the criteria to evaluate the previous test (see below), incompatibility within the order (e.g. ANA together with ANA Reflex), order-priority (routine or urgency), ward-group, rule outcome (see further on), sample-type (e.g. serum or urine) and reporting-comment.

**Fig. 1:**
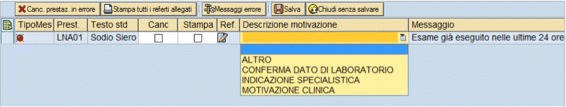
Pop-up prompted to the physician when a rule triggers a Warning with motivation in the order entry system of the IH

The match of a rule triggers one of the following pop-ups:

- *Blocking warning*. A reporting comment is displayed to the physician indicating why the request is blocked. The only allowed action is to cancel the test from the order (i.e. hard stop). An example is an LE request for ANA together with ANA Reflex; the incompatibility between them forces the physician to remove one from the prescription.
- *Warning with motivation*. This warning is similar to a blocking warning, but the physician is allowed to continue the prescription after providing a motivation (i.e. soft stop). To speed a physician’s tasks, predefined motivations were set (specialist request, clinical evidence, laboratory data confirmation, other). An example of a warning with motivation alert from the order entry system at IH is shown in Fig. 1, for a request relative to sodium (clinical chemistry category), which has been already executed in the latest 24 h. An appropriate repetition requires at least 24 h between requests for sodium in the patient’s record.

In cases in which the previous result is valid, a pdf will be generated and submitted to the physician to be evaluated and eventually archived.

### Study design

The study can be considered an observational study addressed to compare the laboratory test ordering and cost trends between two groups: the ‘intervention’ wards where the CDSS was installed and used, and the ‘control’ wards where the CDSS was not installed. The study intended to assess if a decrease had occurred in the test volume and costs in the intervention group with respect to the control one.

The intervention group consisted of the Medicine and Cardiology wards of Cento and Delta Hospitals and the Medicine ward of the IH. The control group consisted of the Post-Acute and Long-term care wards at Cento and Delta Hospitals, the Coronary Intensive Care wards at Cento and Delta Hospitals, and the University Internal Medicine ward at the IH.

In both groups, we included all patients admitted to the wards considered in the study, by comparing a 3-month period before (2014) and after (2015) the intervention. The comparison among wards belonging to the same hospital has been carried on to eliminate the influence of different management strategies of patients (not depending from the CDSS intervention). In order to account for changes over time in the characteristics of patients that might influence the propensity of physicians to order additional tests, some variables such as number of inpatients, patients’ sex and average age, mortality rate and 30-day readmission rate were also recorded. They are shown in Table 2.

**Table 2:** Characteristics of inpatients in the intervention group pre-CDSS installation (2014) and post-CDSS (2015) installation

Variables	2014	2015
Inpatients (N)	1233	1222
Sex female (%)	51.25	51.12
Average age (years)		
*Medicine (Cento)*	75.35	76.80
*Medicine (Delta)*	76.02	77.23
*Medicine (Ferrara)*	73.75	77.75
*Cardiology (Cento)*	69.90	69.30
*Cardiology (Delta)*	72.17	72.76
Mortality rate (%)	10.46	12.03
30-day readmission rate (%)	10.46	10.30

From the clinical data repository the total number of routine requests for eight analytes, executed from September to November 2014 and from September to November 2015 at the Medicine wards of Delta and Ferrara Hospitals, have been extracted to compare the LEs order trend before and after the CDSS installation: activated partial thromboplastin time (aPTT), calcium, blood count, C-reactive protein (CRP), prothrombin time (PT), sodium, urea, free thyroxine (fT4). This set of LEs includes LEs subject to rules based on time and quantitative criteria, and LEs subject to rules based on time criteria only. The total number of examinations was compared between the two time periods in both groups, highlighting the percent difference.

### Measurements and outcomes

This study reviews the effects produced by the introduction of a CDSS in terms of (1) laboratory orders trend (appropriateness/efficiency evaluation), (2) cost trend (economic evaluation), and (3) user satisfaction (organizational evaluation), by extending the early-stage results presented in [11, 12]. It highlights the variations measured before and after the CDSS implementation in both the intervention and control groups.

For the economic evaluation (2), LEs costing data were used to measure the cost reduction from the healthcare system perspective for not performing unnecessary duplicate orders, starting from the cost of each test [13]. The cost of each test is represented by the fee charged for the specific test as per the regional funding mechanism; it does not capture cost savings associated with specimen transport, specimen processing, and the time needed for performing each test. As a proxy of the cost, the use of the charge can be justified because the analysis intends to simply evaluate the impact of this system on the health care organizations, as a result of the reduction in the number of tests performed, rather than possible effects on the hospital cost accounting system and economic efficiency. This enables us to generalize the achieved results in a national context since every laboratory has a unique cost profile for the tests it performs. Each cost profile is the result of different instruments, wage and benefit packages, employee productivity levels, and various costs associated with reagents, supplies, and overheads in a particular laboratory; any economic analysis which includes only reagents and supply costs may underestimate the cost of the laboratory test.

This study did not take into account some indirect costs related to patients on whom tests were performed, such as:

- drugs and costs of performing the tests or follow-up treatments by the patient;
- loss of work productivity related to the patient being admitted to hospital;
- stress related to waiting for test results (intangible costs).

For the organizational evaluation (3), we tried to estimate the users’ response to the CDSS utilization, since these systems often produce significant effects on users’ workflow, efficiency, work structure, and medication safety [14]. Several studies have shown that end-user satisfaction is a critical factor in IT implementation; despite the potential benefits, several attempts to implement CPOE systems have failed, since they met with high levels of user resistance or produced safety problems [8, 15–21]. End-user satisfaction is conceptualized as the affective attitude towards a specific computer application by someone who interacts with the application directly [22]. If users are not satisfied with a computer application, they will tend to avoid it and look for other tools to help them perform their tasks. Some of the barriers to clinicians’ adoption of these systems are usability problems, including human-computer interaction issues causing users’ frustration, time delay in ordering, and workflow disruptions that lead to users’ dissatisfaction with the system. Physicians and nurses constitute two very different types of users: for nurses, ease of use and legibility are often the most important factors, while for physicians, usefulness in terms of efficiency in taking decisions is relevant.

The tools and evaluation models for end-user satisfaction described in the literature range from determining the perceived characteristics of CPOE (for instance, response time) to organizational and clinical aspects, in most cases gathered through questionnaires.

The main study regarding user satisfaction in general settings is by DeLone and McLean [23]; they first elaborated questionnaires in different organizational contexts in order to evaluate system quality in terms of accessibility and ease of use, information quality, usefulness, overall satisfaction, and use of the system with regard to the software and the information presented.

To measure end-user satisfaction after the installation of PROMETEO, a questionnaire based on POESUS [16], but modified according to the specific characteristics of the CDSS, was created and proposed to both nurses and physicians in the involved wards. The questionnaire was tested by a subset of users (staff) involved in the project and updated based on their comments, before submission. The questionnaire responses were tested for internal consistency using the Cronbach’s alpha, whose value resulted of.86.

The pre-CDSS questionnaire allowed at recording users’ satisfaction with the order entry system (CPOE) already in use in all the hospitals, while the post-CDSS questionnaire allowed at capturing users’ satisfaction data with the CDSS built on top of the CPOE, 6 months after its introduction. The questionnaire had 22 closed questions to which the answers were given on a 1 to 5 Likert scale.

## Results

### Cost and test ordering assessment

Table 3 highlights the total number of tests performed, the total cost of the tests, and the corresponding variation measured before and after the CDSS implementation in both the intervention and control groups.

**Table 3:** Total number of tests performed, total cost of the tests and corresponding % variations pre-CDSS implementation and post-CDSS implementation per ward for the intervention and control groups

	Wards	N. tests 2014	N. tests 2015	% tests	Total cost 2014 (euros)	Total cost 2015 (euros)	% costs
**Intervention**	Cardiology	5007	4820	-3.73%	19122.35	17980.35	-5.97%
	Medicine	41499	34040	-17.97%	165002.5	135712.9	-17.75%
	**Total**	46506	38860	**-16.44**%	184124.85	153693	**-16.53**%
**Control**	Post-Acute and Long-term care	3959	4125	4.19%	14950.75	14674.9	-1.85%
	Coronary Intensive Care	2989	2559	-14.39%	13309.5	10643.9	-20.03%
	Medicine (University)	9334	10209	9.37%	41856	46045	10.01%
	**Total**	16282	16893	**3.75**%	70116.25	71363.8%	**1.78**%

Bold entries correspond to the totals

With regard to the tests ordered in the intervention group wards, a total general reduction (-16.44%) over all wards, in each hospital involved, was observed between 2014 and 2015. With regard to the control group, there was a general increase (+3.75%) of tests ordered over all control wards of all hospitals.

When considering the cost of the tests, there was a total reduction (-16.53%) over all wards of all hospitals between 2014 and 2015, versus an increase (+1.78%) in the control group.

### Assessment of specific tests

The percent differences between the number of routine tests executed for eight common analytes at the Medicine wards of Delta hospital, Cento hospital and IH before and after the CDSS implementation are presented in Table 4.

**Table 4:** Percent differences in the number of routine requests for analytes pre-CDSS installation and post-CDSS installation

Test	Delta (Medicine)	Cento (Medicine)	Ferrara (Medicine)
	*Δ*2014−2015*%*	*Δ*2014−2015*%*	*Δ*2014−2015*%*
aPTT	-31.43%	-48.17%	-31.14%
Calcium	-27.51%	-48%	-28.54%
Blood count	-8.75%	-31.32%	-19.84%
CRP	-10.64%	-13.61%	-10.62%
PT	-25.87%	-28.62%	-27.87%
Sodium	-17.46%	-21.87%	-27.17%
Urea	-18.33%	-69.01%	-11.47%
fT4	-17.24%	-32.50%	-18.87%

For all wards experiencing the technological innovation, we found a relevant reduction in the laboratory tests’ volume for all the considered analytes (up to -69%), while there were no statistically significant differences in the characteristics of inpatients between 2014 and 2015 apart from the mortality rate (see Table 2), for which we found a statistically significant difference at the 1% level. The difference between the mortality rates before and after the CDSS intervention could be explained by the complexity of the patients’ health condition. In this regard, if we assume a positive correlation between clinical complexity and resource consumption, the diagnosis-related group (DRG) weight, that is often used as an indicator of the complexity of a patient’s condition, resulted to be higher in 2015 despite the lower number of inpatients.

### Usability assessment

A total of 149 professionals employed in Cento (103 people) and Delta (46 people) Hospitals were involved in the pre-CDSS assessment: physicians (70%) and nurses (30%). On average, nurses’ experience in the use of the technology (3.10 years) was longer than that of the physicians (1.60 years). However, considering the different wards and the same group of professionals (physicians or nurses), a relevant difference in terms of experience has not been detected. A subset of the Delta Hospital’s staff (23 people) was involved in the post-CDSS assessment.

Table 5 reports some individual attributes (gender, tenure, computer experience, education, and occupation) of the interviewees 3 months before (T0) and 6 months after (T6) the CDSS implementation at Delta Hospital. Counte et al. [24] concluded that the impact of these characteristics on the attitude towards computers is unpredictable. In the first questionnaire (T0) the medical staff made up 19.6% and 91% were women. In the second questionnaire (T6) the medical staff made up 13% and 87% were women.

**Table 5:** Characteristics of the interviewees at Delta Hospital 3 months before (T0) and 6 months after (T6) the CDSS implementation

	T0	T6
Interviewees (N)	46	23
Sex female (N)	42	20
Tenure at hospital <1 y	0	0
Tenure at hospital 1-3 y	0	0
Tenure at hospital 4-6 y	1	0
Tenure at hospital 7-10 y	2	3
Tenure at hospital >10 y	43	20
Years of computer experience	2.44	7.79
Degree	17	7
High school diploma	29	16
Physician	9	3
Nurse	37	20

**Fig. 2:**
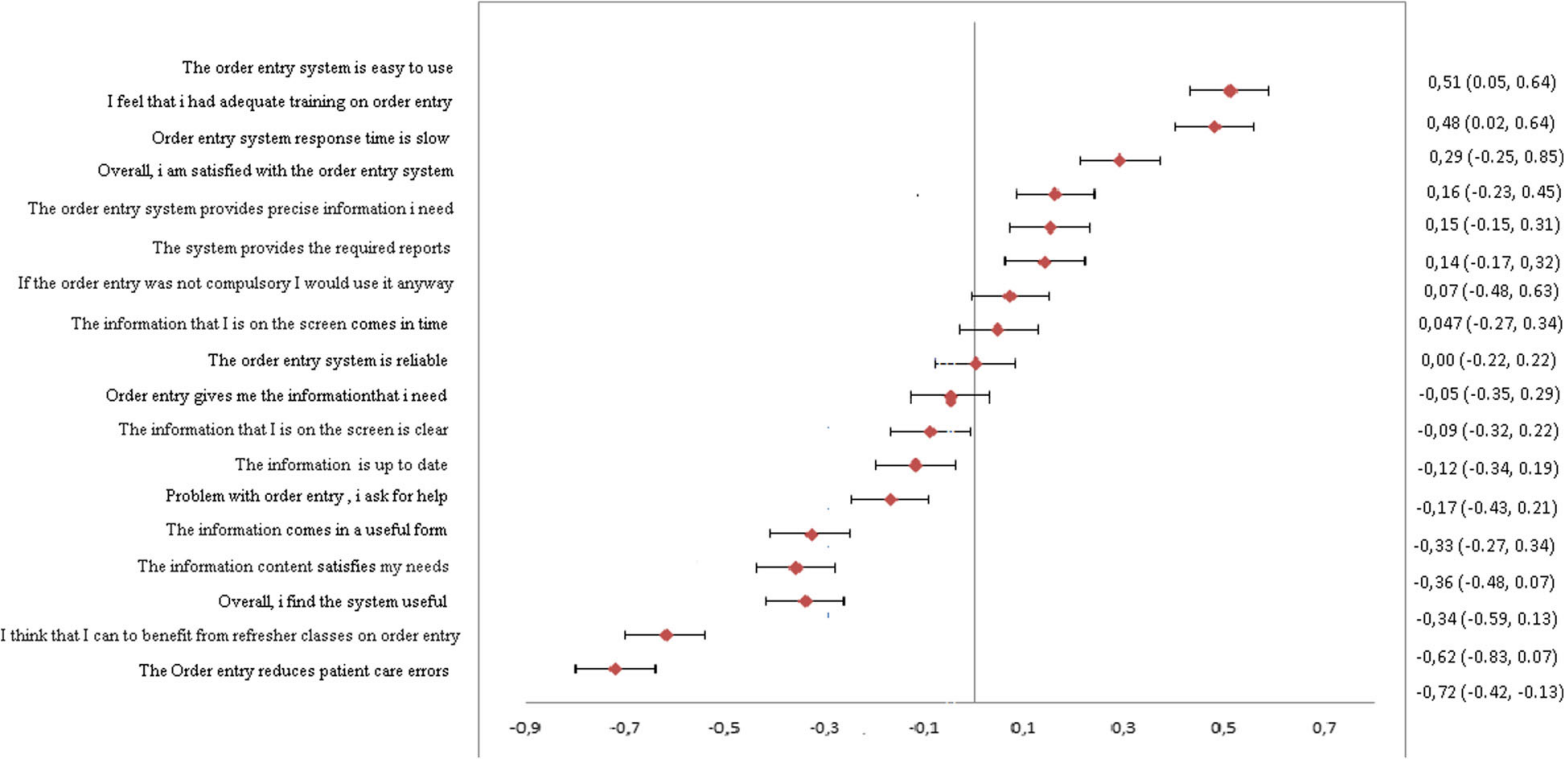
Changes in end-user satisfaction with the CDSS between T0 and T6 at Delta Hospital

The change in user satisfaction between T0 and T6 was evaluated in terms of Cohen’s d and effect size, relative to 18 CDSS characteristics that are illustrated in Fig. 2.

After the introduction of the CDSS, the overall satisfaction did not change significantly, apart from the measures of ease of use (*d* = 0.51) and adequacy of training (*d* = 0.48). The staff gave a positive response toward the information quality, the system quality, and the training classes. Conversely, a higher risk of error and more frequent interactions among the medical personnel were perceived, which indicate some sort of difficulty in adapting to the new system. The interviewees recognized the system’s usefulness in blocking unnecessary tests on the same patient. They noted that the system response time was particularly slow. In two of the wards considered, physicians reported more satisfaction than nurses both with regard to the technology quality and to the quality of the information provided. Based on the characteristics of the CDSS technology linked to its use (usefulness, clarity, updating of the information), the nurses showed a higher level of appreciation than physicians, whereas the characteristics linked to the effectiveness of the system and to work organization (for instance, the error reduction) revealed a lower level of appreciation compared to the pre-CDSS. Finally, as already argued, an analysis of user satisfaction by professionals’ groups revealed no statistically significant difference; and this holds for the general level of satisfaction (*F* = 2.31, *p* > 0.05) and for the attitude to use the technology, although not compulsory (*F* = 3.78, *p* > 0.05).

## Discussion

**Fig. 3:**
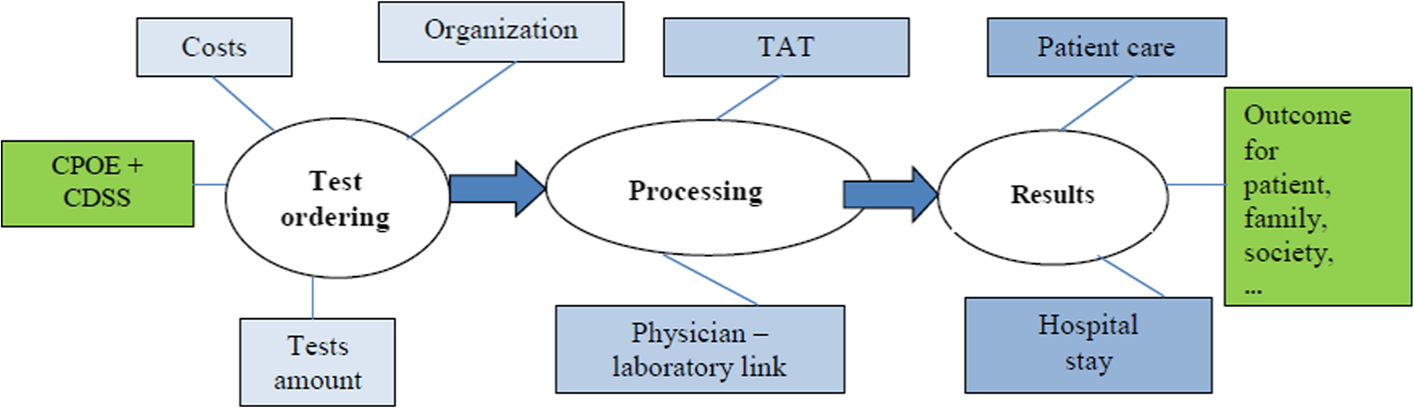
Test ordering workflow and effects

Evidence and studies regarding the appropriateness of laboratory testing show that the issues that determine inappropriateness are multifactorial. Only a multidisciplinary, widely agreed approach would enable us to fully evaluate the effectiveness of possible solutions through projects specifically oriented to achieve a higher accuracy in prescribing tests. Figure 3 shows how a thorough evaluation of the impact of a CDSS should consider not only the direct component related to the laboratory test ordering but also all the related health care issues.

To this end, our study tried to evaluate the overall impact of a decision support system in terms of consequences on costs, on the amount of ordered tests and, finally, on job (re-)organization. The integration of these topics is rarely found in the literature.

Nine studies have assessed the impact of reminders and one study assessed the impact of a reminder targeted to redundant laboratory tests. This last study, conducted by Bates and colleagues [1], showed a statistically significant reduction in test ordering between the intervention and control groups (in the control group, 51% of ordered redundant tests were performed, whereas in the intervention group only 27% of ordered redundant tests were performed). More recently, Chami et al. [3] found that between 6 and 20%, depending on the analyte, of tests ordered were inappropriate based on repeat criteria. There is still insufficient data on the influence of CDSSs on the management of patients with multiple chronic diseases, clinician workload, and length of stay of hospitalized patients [25], even though it is recognized that a CDSS may improve patient safety and, in particular, reduce serious medication errors [26, 27]. In the studied national context, only one other study can be found which analyzed the impact of a computerized alert system based on re-testing intervals in two wards of the University Hospital of Parma; the study found that 22% of test requests violated the criteria of appropriateness and generated an electronic alert [28].

No studies were identified which met the inclusion criteria on the acceptability of a CDSS to physicians or patients [29]. However, different authors [30] agreed on the need for a systematic approach to laboratory and field testing of a CDSS, including the evaluation of effects on health care processes and patient outcomes. Therefore, user acceptance and satisfaction with a CDSS is highly important; if users find that the system does not benefit them, then they will either not use the system or will use it in a suboptimal manner [31].

In our analysis, although the number of inpatients remained constant between 2014 and 2015, we found a 16.44% decrease in the number of performed tests and a 16.53% decrease in the total costs after the introduction of the system in the intervention group. The observed difference in the mortality rate between 2014 and 2015 depended on the different level of severity of the inpatients’ pathologies. In addition, the CDSS was activated for approximately 10% of requests at IH and the warning with motivation alert was bypassed (in order to repeat an examination) in 50% of cases. The solicitation of feedback from the medical staff was also useful for their initial and continued support for this project. Feedback from practice showed that they were generally satisfied with using the software and understood its benefits, but there was less satisfaction with the technical performance in terms of slow response time.

This study should be interpreted in light of its strengths and limitations. We collected data from a limited number of wards from the same health organization, thus the generalizability of the results could be restricted. In the user satisfaction assessment there is probably some dependency in the samples in T0 and T6, since a fraction of the participants filled out the questionnaires in both rounds of data collection. In addition, the question of whether a CDSS is able to improve patient outcomes beyond processes remains open. Because of the study design, we were not able to examine the impact of the CDSS on patients in terms of medication prescription errors or adverse drug events. However, this study, together with a cost analysis and an appropriateness analysis of redundant LEs, provides a first evaluation of the CDSS effects on clinician acceptance of the new technology, for which evidence is still lacking.

The analysis of end-user satisfaction (involving both physicians and nurses) with the software used in an Italian health organization is the first to be carried out in our country. No other study has compared the situation before and after the implementation of a CDSS to highlight adjustment difficulties of the staff in its routine use. Furthermore, to the best of our knowledge, it represents the first Italian project trying to apply ICT resources to test redundancy management in health care on an extensive number of LEs.

## Conclusions

We have evaluated the impact of a CDSS adopted in the prescription phase in some wards of two health care organizations in the province of Ferrara (Italy) to improve organizational performance and to achieve economic savings by advising professionals in real time on the appropriateness of repeating specific routine LEs for inpatients. The effects of the CDSS were assessed in terms of the control of redundant LE requests, cost changes, and end-user satisfaction. We compared the number of LE requests in wards that used a CDSS and wards that did not, over a period of 3 months before (2014) and 3 months after (2015) CDSS installation, and found that 16.44% fewer LE requests were made (7646 across a few wards) after the introduction of the CDSS; in other words, many unnecessary, redundant orders were blocked in wards that had a CDSS and that led to a significant reduction in health care costs (30,400 euros).

## Abbreviations

ANA: Antinuclear antibodies
CDSS: Clinical decision support system
CPOE: Computerized physician order entry
(G)UI: (Graphical) User interface
HbA1c: Glycated hemoglobin
ICT: Information and Communication Technology
IH: Independent Hospital of Ferrara
LE: Laboratory examination
LHA: Local health authority
TSH: Thyroid-stimulating hormone
